# Digitalisierung in der Beratung: Online- und KI-unterstützte Beratungsformate

**DOI:** 10.1007/s11613-023-00803-9

**Published:** 2023-02-06

**Authors:** Silja Kotte, Thomas Webers

**Affiliations:** 1 Technische Hochschule Aschaffenburg, Würzburger Str. 45, 63743 Aschaffenburg, Deutschland; 2 Paulstr. 9, 53111 Bonn, Deutschland

Begünstigt durch die Covid-19-Pandemie und die rasante Entwicklung der Informations- und Kommunikationstechnologien befinden sich die Arbeitswelt und Organisationen in einem grundlegenden Wandel (Foster et al. 2019; Wang et al. 2021), der oft durch das Akronym VUKA (Volatilität, Unsicherheit, Komplexität, Ambiguität) beschrieben wird. Das OSC-Heft 4/2022 hat die Auswirkungen der Digitalisierung auf virtuelles Arbeiten und Führung auf Distanz beleuchtet.

Die Digitalisierung beeinflusst jedoch nicht nur die Zusammenarbeit in Organisationen selbst. Einerseits steigt durch die VUKA-bedingten Veränderungen der Bedarf an kontinuierlichem personalisiertem Lernen und Entwicklung deutlich. Organisationen müssen in die Fähigkeiten ihrer Mitarbeitenden investieren, sich selbst zu steuern, mit verteilter Führung umzugehen und flexibel auf sich verändernde Arbeitsanforderungen zu reagieren. In diesem Zusammenhang ist Coaching als kollaboratives, reflexions- und zielorientiertes Beratungsformat zu einem zentralen Instrument des Personalentwicklungs-Portfolios der meisten Unternehmen geworden. Andererseits haben sich durch den Digitalisierungs-Schub auch die Möglichkeiten des digitalen Lernens und damit die Personalentwicklungs-Praktiken vieler Unternehmen stark erweitert. Arbeitgeber bieten zunehmend Online-Lernmöglichkeiten an, die von Arbeitnehmer:innen auch zunehmend genutzt werden, z. B. durch Virtual-Reality-Simulationen, elektronische Chat-Plattformen und Video-Konferenz-Systeme (World Economic Forum 2020). Der E‑Learning-Markt etwa hat sich in den letzten 20 Jahren von einem Nischenmarkt zu einem globalen Markt entwickelt.

Lange haben sich Coaches hierzulande der Digitalisierung eher verweigert. Es wurde behauptet, „richtiges“ Coaching funktioniere nicht online (Middendorf 2016; Fröhlich und Webers 2020). Das verwundert, führt man sich u. a. die über 20-jährige Erfolgsgeschichte der Telefonseelsorge vor Augen. Das e‑beratungsjournal.net, die Zeitschrift für Onlineberatung & computervermittelte Kommunikation, berichtet nun schon im 19. Jahrgang über diese Entwicklungen. Man mag über die Gründe für die Zurückhaltung und den beachtlichen Zeitverzug spekulieren. Die Frage der Marktpositionierung als hochpreisiges Dienstleistungsangebot, das einen exquisiten Rahmen benötigt, kann als ein Grund neben anderen angeführt werden. Letztlich ist es jedoch müßig, solchen Fragen nachzugehen – zumal es ja auch schon länger löbliche Ausnahmen gibt, auf die hinzuweisen wäre, wenn diese in der Praxis auch weniger Widerhall fanden (Geißler 2008, 2018; Heller et al. 2018; Berninger-Schäfer 2018; Wegener et al. 2020).

Doch die Lockdown-Situation während der Corona-Pandemie hat die Lage radikal verändert. In diesem Zuge hat sich auch Coaching gewandelt. Coaching und verwandte Beratungsformate finden nicht mehr in erster Linie face-to-face in Präsenz statt, sondern in verschiedenen Settings, mit verschiedenen Methoden und über verschiedene Kanäle (z. B. online/digital oder hybrid; video- oder chatbasiert; vgl. Pascal et al. 2015; Middendorf 2021).

In Folge der technologischen Entwicklungen wird auch künstliche Intelligenz (KI, im Englischen: artificial intelligence, AI) von Unternehmen zunehmend eingesetzt. KI in ihrer grundlegendsten Form bezeichnet die (Teil‑)Automatisierung von Arbeiten, die traditionell von Menschen erledigt werden. Und mehr noch: Intelligentes menschliches Problemlösungsverhalten soll durch Maschinen nachgebildet oder simuliert werden. Unternehmen nutzen KI bereits für eine Vielzahl von Aufgaben, z. B. zur (Unterstützung bei der) Beurteilung und Auswahl von Mitarbeitenden, Finanz- und Versicherungsberatung, Planung komplexer Logistik- und Prognoseaufgaben oder Diagnose und Behandlungsvorschlägen. KI-Investitionen von Unternehmen weltweit sind in den letzten Jahren drastisch gestiegen – von 5,23 Mrd. Dollar im Jahr 2013 auf 176,47 Mrd. Dollar im Jahr 2021 (Zhang et al. 2022).

Damit gewinnt auch die Anwendung von KI in der Beratung zunehmend an Interesse. Zwar ist es unwahrscheinlich, dass KI in absehbarer Zukunft menschliche Coaches vollständig ersetzen wird („general/strong AI“), etwa um menschliches Einfühlungsvermögen zu reproduzieren – wie das manche befürchten. Dennoch spielt „narrow/weak AI“ beim Coaching bereits jetzt eine Rolle, indem sie spezifische Coaching-Aufgaben übernimmt und insbesondere bei Chatbot-basiertem oder -unterstütztem Coaching (z. B. Graßmann und Schermuly 2021; Terblanche 2020) eingesetzt wird. Chatbots nutzen dialogbasierte, automatisierte Software, die menschliche Unterhaltungen imitiert. Chatbots können regelbasiert sein, also vordefinierten Entscheidungsbäumen folgen, z. B. indem sie verschiedene Fragen und Übungen auf der Grundlage der Entscheidungen und Reaktionen von Coachees anbieten. Sie können auch KI-basiert oder eine Kombination aus beidem sein. KI-basierte Coaching-Chatbots beobachten das Verhalten der Coachees, lernen daraus, sagen es voraus und führen einen adaptiven Dialog, um Lernen und Zielerreichung zu unterstützen. Im Bereich der Psychotherapie ist SimCoach ein solches niedrigschwelliges Angebot, mit dem traumatisierte amerikanische Veteranen (z. B. aus dem Afghanistan-Einsatz) erreicht werden konnten, die sich sonst nur selten Hilfe holten: Man kann dort anonym mit einem Avatar in Kontakt treten, der Hilfestellungen anbahnen kann (vgl. das Video unter https://www.youtube.com/watch?v=2bsMESwBeyg).

Derzeit befindet sich Chatbot-Coaching noch in einem frühen Entwicklungsstadium und kombiniert in der Regel regelbasierte Chatbots mit KI-Elementen. Chatbot-Coaching bietet einige Potenziale, u. a. Coaching für Mitarbeitende in der Breite zugänglich zu machen, sodass Coaching leichter skaliert werden und organisationale Wirkungen entfalten kann. Darüber hinaus können Chatbots menschliche Coaches unterstützen, indem sie einfache, konkrete Aufgaben (z. B. Anleitung von Coaching-Übungen, Nachhalten mittels Remindern) innerhalb von Coachingprozessen übernehmen können.

Die technologischen Entwicklungen erfordern auch im Hinblick auf die Forschung zu Coaching, Supervision und verwandten Beratungsformaten neue Herangehensweisen. Die Bezüge zur Trainings- und Psychotherapieliteratur und etablierten Theorien aus Psychologie, Soziologie und verwandten Disziplinen müssen erweitert werden um Perspektiven, die Theorien und Befunde aus jüngeren Forschungsfeldern wie der Medienpsychologie und der Mensch-Maschine-Interaktion integrieren. Die Forschung zu digitalen Coaching-Ansätzen ist hier bislang noch zu wenig theoretisch unterfüttert, die Forschung zu KI-gestütztem Coaching oft hauptsächlich konzeptioneller Natur. Die wenigen empirischen Studien, die es bisher zu Chatbot-Coaching gibt, lassen zudem den organisationalen Kontext von Coaching eher außer Acht.

Im Mittelpunkt des vorliegenden Heftes steht daher die Frage, wie neue technologische Entwicklungen in Coaching, Supervision und Konfliktberatung integriert werden und wie sie diese verändern (können). Dabei geht es sowohl um digitale Formen der Beratung, u. a. um wirksames Handeln in digitalen Settings, konkrete Techniken und Methoden sowie organisationale Einflussfaktoren als auch um Chatbot-basiertes oder -unterstütztes Coaching. Ziel ist es, den Leser:innen Impulse zu geben, ihre eigene Beratungspraxis im Umgang mit neuen Technologien zu reflektieren, neugierig auf die damit verbundenen Möglichkeiten zu machen und deren Grenzen einschätzen zu können. Die Parole kann und darf nicht lauten, „den Geist zurück in die Flasche zu sperren“. Coaches, Personalentwickler:innen und Organisationen müssen sich mit den (neuen) Möglichkeiten aktiv auseinandersetzen. Es gilt, kompetent zu werden, damit ein verantwortliches Handeln möglich wird. Damit ist – neben den technologischen – auch die Berücksichtigung ethischer Aspekte gemeint, die über Fragen des Datenschutzes deutlich hinausgeht (Misselhorn 2021). Die Zukunft des Coachings, so zeigen Schermuly et al. (2021), wird von neuen Anforderungen und Chancen geprägt sein. Die beratende Zunft, die von ihrer Klientel Offenheit und Veränderungsbereitschaft fordert und erwartet, kann hier als Vorbild vorangehen.

Die Beiträge zum Themenschwerpunkt dieses Heftes beschäftigen sich mit Besonderheiten, erfolgsversprechenden Praktiken und Herausforderungen von Online-Coaching, Online-Supervision und virtueller (Konflikt‑)Beratung sowie einer Einführung in das Chatbot-basierte Coaching und dessen Veranschaulichung anhand von Anwendungsbeispielen.

Der Beitrag über Erfolgsfaktoren im Online-Coaching der Autor:innengruppe um *Harald Geißler* fokussiert auf Online-Coaching (im Audio- oder Videoformat), das mit zusätzlichen Online-Coaching-Problemlösungsmedien (z. B. avatarbasierte 2D‑/3D-visuelle Coaching-Tools) angereichert wird. Dabei steht die Frage im Mittelpunkt, welche konkreten Online-Coaching-Praktiken erfolgsträchtig scheinen. Auf der Grundlage coaching- und medientheoretischer Überlegungen wurden auf Video aufgezeichnete Online-Coachings analysiert und induktiv Online-Erfolgsfaktoren herausgearbeitet. Diese werden anhand von Beispielen erläutert und dann in Bezug zu Medientheorien gesetzt. Schließlich wird eine Systematik dargestellt, wie bei den verschiedenen Online-Erfolgsfaktoren elektronische Coaching-Kommunikations- und Problemlösemedien in Bezug gesetzt werden können zu psychischen Verarbeitungsprozessen (die die Autor:innen als „psychische Coaching-Problemlösungsmedien“ bezeichnen).

Der Beitrag von *Marleen Birkmann* basiert auf Experteninterviews mit Berater:innen, die über Erfahrung im virtuellen Konfliktmanagement für Teams verfügen. Die Autorin gibt zunächst einen kurzen Überblick in den Stand der Literatur zum Konfliktmanagement in Teams und speziell in virtuellen Teams. Auf der Grundlage der Interviews wird herausgearbeitet, welche Vorbereitung für virtuelles Konfliktmanagement nötig ist, welche Interventionsmöglichkeiten für die Konfliktbearbeitung sich bieten, welche Herausforderungen durch die virtuelle Umgebung entstehen, wie die Mediennutzung (im Gegensatz zu einem Präsenzsetting) Teammitglieder und Berater:innen beeinflusst und welche Grenzen und Hindernisse sich für die Konfliktbearbeitung zeigen.

Der dritte Hauptbeitrag beschäftigt sich mit Chatbots als einer der maßgeblichen technologischen Innovationen der letzten Jahre. Diese Technologie kann den Coaching-Prozess selbst unterstützen und begleiten. Ein Chatbot kann als virtueller Assistent Klient:innen z. B. durch standardisierbare Prozesse wie Auftrags- oder Anliegenklärung, Hausaufgaben oder Evaluierungen führen. Der Beitrag von *Vanessa Mai *und* Rebecca Rutschmann* gibt einen Überblick über aktuelle Entwicklungslinien von Chatbots im Coaching. Es werden Einsatzfelder in Unternehmen, Organisationen und Hochschulen skizziert sowie Potenziale und Herausforderungen von Coaching-Chatbots benannt. Die Konzeption und die Entwicklung von Chatbots gelingen insbesondere als interdisziplinärer, co-kreativer und iterativer Prozess. Der Beitrag schließt mit einem Ausblick auf die Zukunft von Chatbots im Coaching.

In einem Praxisbeitrag reflektiert *Volker Jörn Walpuski* seine Erfahrungen mit der Videokonferenzsoftware Microsoft Skype for Business® für Gruppensupervisionen. Dabei arbeitet er Besonderheiten der Software, der organisationalen Rahmenbedingungen der Skype-Supervision bei Landes- und Bundesbehörden sowie der Dominanz der IT für das supervisorische Setting heraus. Er erläutert, inwiefern die organisationalen Setzungen stärker als in präsenten Settings die Supervision beeinflussen und die Supervisionsgruppe – sowohl Supervisor wie Supervisand:innen – depotenzieren, und weist auf den damit einhergehenden Mehraufwand an Kontraktierung hin.

*Janette Wendt* fokussiert in ihrem Beitrag speziell auf Kreative Methoden in der Online-Supvervision. Auf der Basis von Interviews mit online tätigen, in erlebnisaktivierenden Verfahren ausgebildeten Supervisor:innen erläutert sie, wie kreative Beratungsmethoden online konkret umgesetzt werden können. Sie beschreibt Methoden zur Eröffnung einer Online-Sitzung, verschiedene Varianten soziometrischer Verfahren, den Einsatz kreativer Medien (u. a. symbolische Arbeit mit Gegenständen, Skulpturen und Einsatz des Körpers), Psychodramatechniken (u. a. Rollentausch, Doppeln), Aufstellungsarbeit und Formen des Playback-Theaters.

Der Praxisbeitrag von *Vanessa Mai* und *Rebecca Rutschmann* stellt zwei Anwendungsbeispiele für den Chatbot-Einsatz im Coaching vor. Zunächst erhält die Leserschaft Einblicke in die Entwicklung des StudiCoachBots der TH Köln, der Reflexionsprozesse bei Studierenden zu Prüfungsangst anregt. Anschließend wird das Startup evoach vorgestellt. Das Unternehmen bietet eine Arbeitsumgebung an, mittels derer maßgeschneiderte Chatbots entwickelt und von Coaches genutzt werden können. Anhand konkreter Anwendungsfälle wird deutlich, wie der Einsatz von Chatbots den Coaching-Prozess bereichern kann.

Außerhalb unseres Themenschwerpunkts geht es um Entwicklungs- und Veränderungsprozesse. Wie können Entwicklungs- und Implementierungsprozesse in der Sozialen Arbeit verlaufen und welche Aspekte sind für das *Gelingen* dieser Prozesse zu berücksichtigen? Die Autor:innengruppe um *Ursula Hochuli Freund* stellt wichtige Ergebnisse eines kooperativ angelegten Forschungs- und Entwicklungsprojekts vor. Das Ergebnis ist ein neues, integriertes Phasenmodell – und die wichtige Erkenntnis, dass Veränderungsprozesse deutlich langfristiger zu konzipieren sind als bislang üblich. Zudem ist es von Vorteil, prozesshaft Entwicklung und Implementierung stark zu verzahnen.

*Knut Hüneke* beschreibt in seinem Praxisbericht die Beteiligung der Beschäftigten in einem Change-Management-Prozess in den Waldkliniken Eisenberg, wo er für das Prozess- und Projektmanagement zuständig war und über zehn Jahre den Neubau des Bettenhauses begleitet hat. Zentral war dabei die gemeinsame Vision: eine qualitativ hochwertige Versorgung, zufriedene Patient:innen, zufriedene Beschäftigte – und die finanzielle Tragfähigkeit. Das vom Stararchitekten Matteo Thun geplante Haus wird inzwischen zu den besten Kliniken Deutschlands gezählt.

Das Heft schließt mit einem Diskurs von *Ferdinand Buer* als Resonanz zum Themenschwerpunkt Coachingforschung im Heft 3/22. Er unterscheidet die drei Wissenssorten Orientierungswissen, das er der Philosophie, Erklärungswissen, das er der (Human‑)Wissenschaft, und Erfahrungswissen, das er der professionellen Praxis zuordnet. Er postuliert, dass diese Unterscheidung der Wissensarten dazu beitragen kann, dass ein Dialog über Coaching und Supervision zwischen der Scientific und der Professional Community gelingen kann, wenn sie zudem erkennen, dass es über den bilateralen Dialog hinaus auch einer philosophischen Reflexion bedarf.
